# Bioassay Guided Fractionation Protocol for Determining Novel Active Compounds in Selected Australian Flora

**DOI:** 10.3390/plants11212886

**Published:** 2022-10-28

**Authors:** Janice Mani, Joel Johnson, Holly Hosking, Beatriz E. Hoyos, Kerry B. Walsh, Paul Neilsen, Mani Naiker

**Affiliations:** 1 School of Health, Medical and Applied Sciences, Central Queensland University, Bruce Hwy, North Rockhampton, QLD 4701, Australia; j.johnson2@cqu.edu.au (J.J.); h.hosking2@cqu.edu.au (H.H.); b.hoyosortiz@cqumail.com (B.E.H.); k.walsh@cqu.edu.au (K.B.W.); p.neilsen@cqu.edu.au (P.N.); m.naiker@cqu.edu.au (M.N.); 2 Institute of Future Farming Systems, Central Queensland University, Bruce Hwy, North Rockhampton, QLD 4701, Australia

**Keywords:** bioassay guided fractionation, antioxidant capacity, total phenolic content, anticancer activity

## Abstract

A large variety of unique and distinct flora of Australia have developed exceptional survival methods and phytochemicals and hence may provide a significant avenue for new drug discovery. This study proposes a bioassay guided fractionation protocol that maybe robust and efficient in screening plants with potential bioactive properties and isolating lead novel compounds. Hence, five native Australian plants were selected for this screening process, namely *Pittosporum angustifolium* (Gumbi gumbi), *Terminalia ferdinandiana* (Kakadu plum, seeds (KPS), and flesh (KPF)), *Cupaniopsis anacardioides* (Tuckeroo, seeds (TKS) and flesh (TKF)), *Podocarpus elatus* (Illawarra plum, seeds (IPS) and flesh (IPF)) and *Pleiogynium timoriense* (Burdekin plum, seeds (BPS) and flesh (BPF)). The methanolic extracts of the plants samples were analysed for Total phenolic content (TPC) and antioxidant capacity measure by FRAP. The highest values were found in the KPF which were 20,847 ± 2322 mg GAE/100 g TPC and 100,494 ± 9487 mg TXE/100 g antioxidant capacity. Extracts of GGL was deemed to be most potent with complete cell inhibition in HeLa and HT29, and about 95% inhibition in HuH7 cells. Comparative activity was also seen for KPS extract, where more than 80% cell inhibition occurred in all tested cell lines. Dose-dependent studies showed higher SI values (0.72–1.02) in KPS extracts than GGL (0.5–0.73). Microbial assays of the crude extracts were also performed against five bacterial strains commonly associated with causing food poisoning diseases were selected (Gram positive—*Staphylococcus aureus* and Gram negative—*Escherichia coli*, *Salmonella typhi* and *Pseudomonas aeruginosa* bacteria). KPF extracts were effective in suppressing microbial growth of all tested bacterial strains except for *P. aeruginosa*, while TKS and TKF were only slightly effective against *S. aureus.* Due to the potential of the GGL crude extract to completely inhibit the cells compared to KPS, it was further fractionated and tested against the cell lines. HPLC phenolic profiling of the crude extracts were performed, and numerous peak overlaps were evident in the fruit extracts. The KPF extracts demonstrated the strongest peaks which was coherent with the fact that it had the highest TPC and antioxidant capacity values. A high occurrence of t-ferulic acid in the GGL extracts was found which may explain the cytotoxic activity of GGL extracts. Peaks in KPS and KPF extracts were tentatively identified as gallic acid, protocatechuic acid, 4-hydroxybenzoic acid and syringic acid and possibly ellagic acid. HPLC time-based fractionation of the GGL extract (F1–F5) was performed and Dose dependent cytotoxic effects were determined. It was construed that F1, having the highest SI value for HeLa, HT29 and HuH7 (1.60, 1.41 and 1.67, respectively) would be promising for further fractionation and isolation process.

## 1. Introduction

Plants produce several phytochemicals as defense mechanisms to cope with environmental stressors. The largest part of Australia is desert or semi-arid, with only the south-east and south-west corners having a temperate climate and moderately fertile soil. With Australia’s plethora of floral species which are adapted to the harsh environment, there remains opportunities to investigate the bioactivity and chemical properties of their phytochemicals. Furthermore, Australian Aboriginal and Torres Strait Islander people have depended on traditional plant-based medicines for centuries. However, Australian plant species and traditional plant-based remedies currently being utilized and practiced by indigenous communities remain unexplored from a scientific perspective.

Several different phytochemicals such as alkaloids, flavonoids, phenolic glucosides, saponins and tannins have been identified in plants to have antioxidant and therapeutic properties [1]. Bioactive compounds derived from certain Australian plants have already been connected to significant health benefiting outcomes and the phytochemistry and bioactivity of these native Australian plants, have been collated and documented in our previous review paper [2].

To investigate the bioactivity of phytochemicals and to discover novel compounds occurring in the vast variety of plant species, development of an efficient and robust technique is required. A typical approach to obtain pure isolates from plant origin is bioassay-guided fractionation. Bioassay-guided fractionation may be defined as a technique for profiling and screening of plant extracts for bioactive compounds with potential sources of new bio-based drugs [1]. The fractionation procedure enables the screening and purification of natural products in the plant extracts more efficiently. This process of separating components in plant extracts using chromatographic separation techniques leads to the isolation of pure compounds which are biologically active in preclinical in vitro experiments.

**Figure 1:**
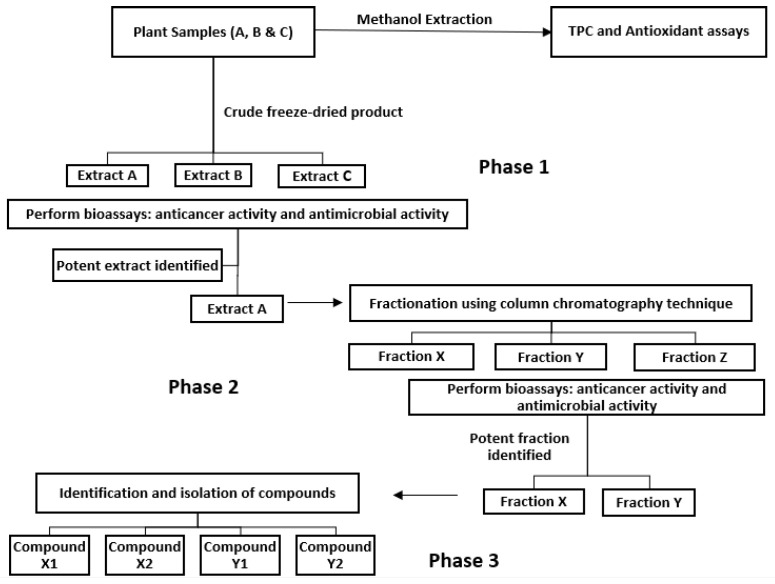
Bioassay guided fraction protocol design [3]. Reproduced under Creative Commons CC-BY-NC-ND license.

The present study aims to design a robust bioassay-guided protocol design and test its efficiency and effectiveness. The proposed design of the protocol is given in Figure 1. It first aims to investigate the phenolic content and antioxidant capacity in methanolic extracts of plants, namely *Pittosporum angustifolium* (Gumbi gumbi), *Terminalia ferdinandiana* (Kakadu plum), *Cupaniopsis anacardioides* (Tuckeroo), *Podocarpus elatus* (Illawarra plum) and *Pleiogynium timoriense* (Burdekin plum). Subsequently, plants exhibiting high phenolic content and antioxidant capacity will be selected for in vitro anticancer and antimicrobial bioassays, which is Phase 1 of the design protocol (Figure 1) and has been previously reported in our recent publication [3]. The extracts exhibiting bioactivity will then be selected for Phase 2, which involves fractionation using column chromatographic techniques and performing bioassays on the separated fractions of the extracts (Figure 1).

## 2. Results

### 2.1. Total Phenolic Content (TPC) and Antioxidant Capacity

Among the five different native Australian plants studied, KPF exhibited the highest antioxidant capacity and TPC value (Table 1). Notable values were also seen in BPF and TKF and GGL. High antioxidative capacity and total phenolic content often dictates the bioactive potential of a plant matrix [2,4,5]. Hence, it was postulated that KPF would show high bioactivity in the anticancer and antimicrobial bioassays. Moreover, Kakadu plum and Illawarra plum have previously been shown to exhibit significant in vitro antioxidant, anti-inflammatory, and proapoptotic anticancer activity [6,7,8,9,10]. Furthermore, Kakadu plum fruit have the highest recorded concentrations of ascorbic acid of any fruit in the world [11].

However, the flesh and seeds of these fruits have not been separately tested for their phenolic content, antioxidant capacity, and bioactivity. Data obtained herein shows that the TPC and FRAP content are significantly different (*p*-value < 0.05) between the flesh and seeds of the plum samples. Although, the seeds have lower TPC and antioxidant capacity compared to the flesh and are often discarded, they still have valuable amounts of phenolic compounds and antioxidants which may be harnessed for therapeutic or nutritional uses.

### 2.2. Cytotoxic Activity of Crude Extracts

#### 2.2.1. Crude Extracts Cytotoxicity

**Figure 2:**
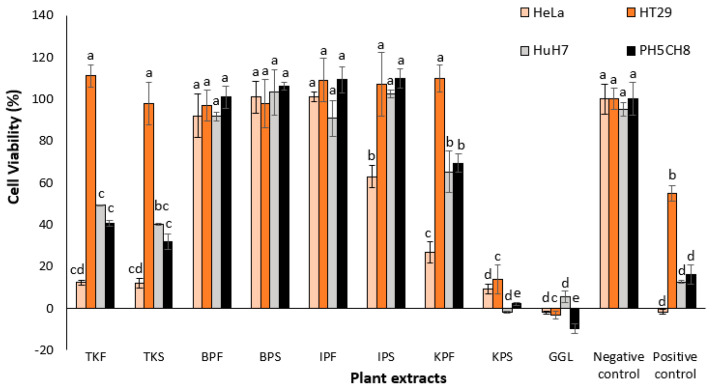
Percentage cell viability of cell lines treated with plant extracts at 500 µg/mL concentration. One-way ANOVA test indicated a significant difference (*p*-value < 0.05) in cytotoxicity between the different plant extracts for the same cell line, denoted by different letters on the respective bars. Negative control: cells without treatment; positive control: cells treated with 50 µg/mL cisplatin (chemotherapy drug).

**Figure 3:**
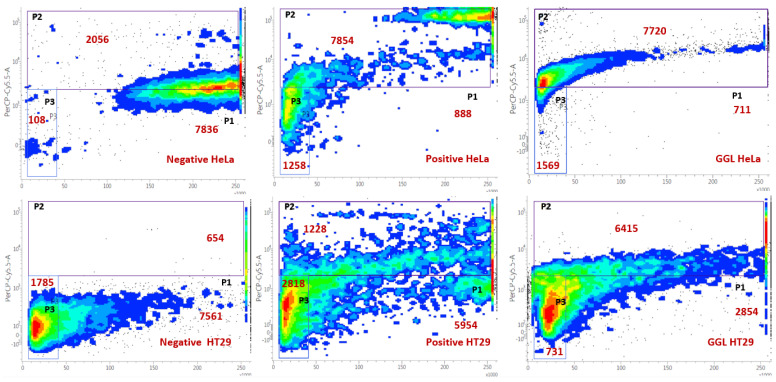

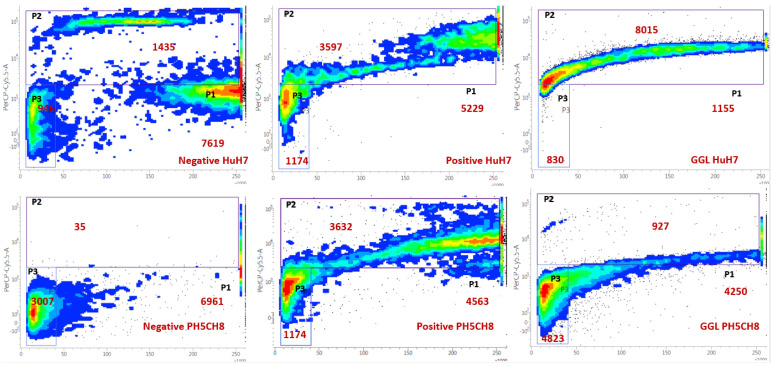
Flow cytometry image depicting cell viability of HeLa, HT29, HuH7 and PH5CH8 cells tested against negative and positive controls, and GGL extracts. The regions P1, P2 and P3 represent live cells, dead cells and debris, respectively.

Each plant extract was screened for in vitro cytotoxic properties against HeLa, HT29 and HuH7 cancer cell lines and PH5CH8 (normal epithelia cells) using the MTS assay and is presented in Figure 2. Highest cytotoxicity against cancer cell lines was noted in GGL extracts which showed complete (100%) cell inhibition in HeLa, HT29 and PH5CH8, and about 95% inhibition in HuH7 cells. Comparative activity was also seen for KPS extract, where more than 80% cell inhibition occurred. Tuckeroo samples (TKF and TKS) and KPF showed similar activity (>70% cell inhibition against HeLa cells) and higher selectivity due to less toxicity against the PH5CH8 cells. The tuckeroo samples however, had greater toxicity against the HuH7 cells (>50% cell inhibition) compared to KPF (35% cell inhibition). Generally, the HeLa cells were found to have greater susceptibility to the extracts compared to the other cell lines tested. The 7-AAD flow cytometry cell viability analysis also confirmed the inhibitory effects of the above mentioned extracts against the cell lines tested. Flow cytometry images of GGL extracts compared to the negative and positive controls against HeLa, HT29, HuH7 and PH5CH8 are shown in Figure 3. The flow cytometry images of the GGL extracts were visually comparable to those of the positive control for all the cell lines tested. This viable and nonviable cell distribution pattern and gating of the cell population of the flow cytometry image was similar to a previous study analyzing the cell viability of HK2 (human kidney 2) cryopreserved cells [12].

#### 2.2.2. Inhibitory Concentrations and Selective Index

The degree of selectivity of an extract or compound can be expressed by its selectivity index (SI) value [13]. Hence, dose-dependent studies were performed on the most promising cytotoxic extracts (GGL and KPS). The IC_50_ and SI values of GGL and KP obtained are presented in Table 2. Comparatively, KPS extracts showed higher SI values (0.72–1.02) than GGL (0.5–0.73). A higher SI value is indicative of protection to a normal cell whilst increased toxicity towards the cancerous cells [14]. Moreover, SI values greater than 2 of a compound indicate selective toxicity towards cancer cells, whereas compounds with SI less than 2 is considered to give general toxicity including cytotoxicity in normal cells [13]. The SI values of both GGL and KPS showed values which were lower than 2, thereby suggesting their use as anticancer remedies to be unfavorable. Nonetheless, due to the potential of the GGL crude extract to completely inhibit the cells compared to KPS, it was further fractionated and tested against the cell lines. It was postulated that perhaps the fractions of the GGL crude extract may present more favorable IC_50_ and SI values, with the ultimate hope of identifying compound(s) which may be responsible for the cytotoxic property of GGL crude extracts. Additionally establishing the chemical structure and mechanism of action of compounds which kill normal cells could potentially be useful for designing new anti-cancer drugs, as it will indicate which molecular pathways need to be avoided to avoid killing normal cells.

**Figure 4:**
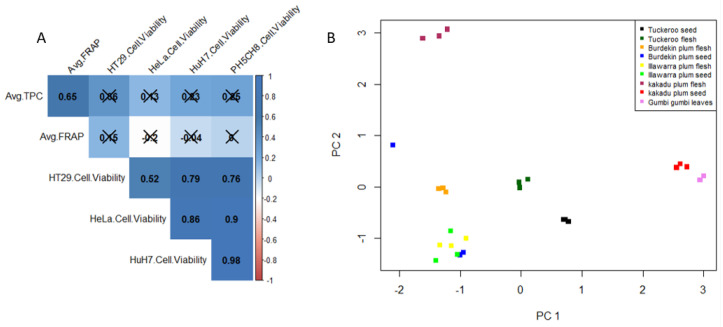
(**A**) Correlation plot; (**B**) PCA scores plot.

Overall, a correlogram of the phytochemical and cytotoxicity data of the plant extracts was obtained as given in Figure 4A. It was construed that whilst TPC and FRAP antioxidant showed significant positive correlation (0.65), no correlation was evident between either TPC and the cytotoxic activity nor between FRAP and the cytotoxic activity. Previous studies have shown that higher TPC and antioxidant capacity potentially result in higher cytotoxicity [2,15,16]. On the contrary, some studies suggest that a negative correlation may be due to differences in interaction of polyphenols, availability and nature (polar or nonpolar) of compounds with antioxidant capacities [3,17,18]. Moreover, a PCA scores plot of the phytochemical and cytotoxic data along the x-axis shows variability not only amongst the plant species but in some cases between the flesh and seeds (BPF and BPS, and KPF and KPS) (Figure 4B. The distribution of KPS and GGL extracts are in close proximity to each other, which may explain their similar cytotoxic behavior as shown in Figure 4B.

### 2.3. Antibacterial Activity

**Figure 5:**
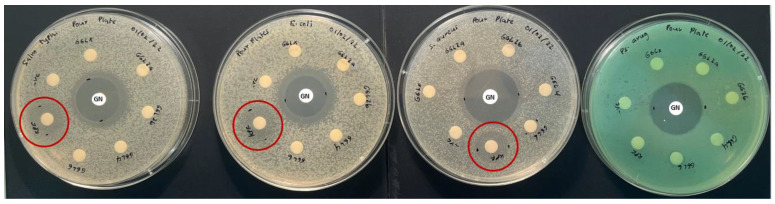
Kakadu plum flesh antibacterial activity.

The antibacterial activity of the selected plant species was investigated against food poisoning bacteria using the disc diffusion method, with the results presented in Table 3. The results revealed that only the KPF extracts were potentially effective in suppressing microbial growth of all tested bacterial strains apart from *P. aeruginosa*, although it did show weaker activity than the positive control (Figure 5). Additionally, TKS and TKF were only slightly effective against *S. aureus.* Other plants extracts showed no antibacterial activity against the pathogenic bacterial strains at the tested concentration.

Previous studies have also demonstrated KPF extracts ability to inhibit the growth of both Gram-positive and Gram-negative bacteria [10,11,19]. Particularly, inhibitory effects against *E. coli* and *S. aureus* was noted at concentrations between 3.1–15.6 mg/mL [11]. Although Kakadu plum has shown exceptionally high ascorbic acid content, this compound alone may not be responsible for the broad antibacterial activity. Studies have shown ascorbic acid to have weak antibacterial activity towards *E. coli* and *S. aureus* [11,17,20]. Hence, it is more likely that the interaction between ascorbic acid and polyphenols is prompting the antibacterial activity in this fruit. Akter et al. (2021) proposed that the Kakadu plum extracts possibly interfere with the bacterial cell walls and induce lysis or the presence of phenolic compounds and other lower molecular weight compounds such as K^+^ and PO_4_^3−^ may also be enhancing the loss of other intracellular molecules such as proteins, DNA, RNA, and other higher molecular weight materials.

**Figure 6:**
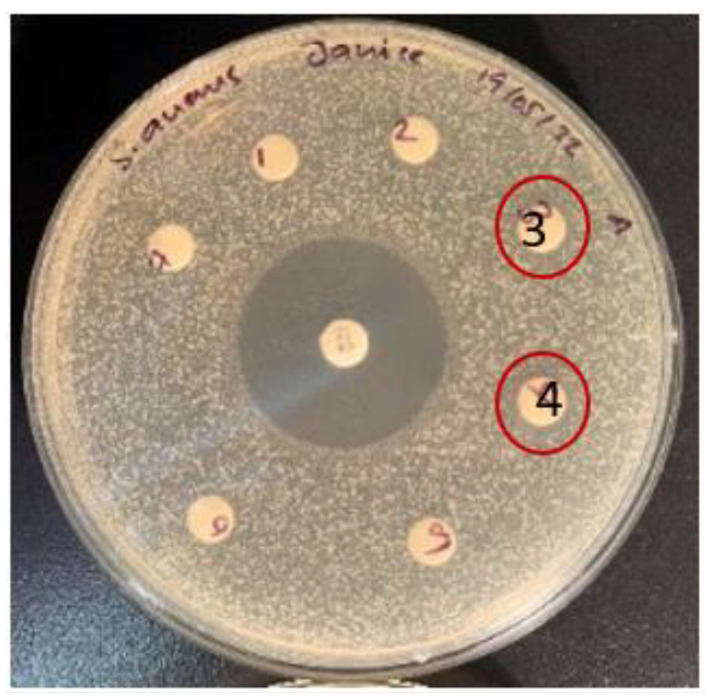
Tuckeroo plum flesh (3) and seeds (4) antibacterial activity plates.

Although some phytochemical studies [21,22] and cytotoxicity studies [16] of tuckeroo fruit exists, no substantial studies report the antibacterial activity of this fruit. This study may be the first to report some antibacterial activity in the flesh and seed of this fruit (Figure 6). The zone of inhibitions of the extracts at 20 mg/mL are quite small hence future dose response studies to calculate IC_50_ values is suggested.

### 2.4. HPLC Profiling and Characterization of the Crude Extracts

**Figure 7:**
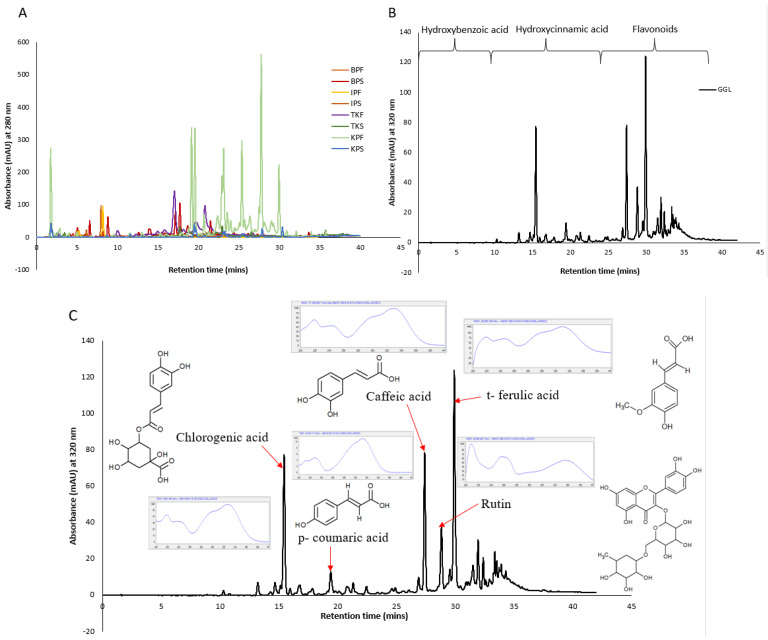
HPLC phenolic profile of the fruits (**A**) and GGL (**B**) methanolic extracts. Tentative peak identification of GGL extracts (**C**).

HPLC phenolic profiling of the crude extracts were performed, and the chromatograms obtained are given in Figure 7 (Panel A and B). With reference to retention time, the elution of polyphenols can be generalized as hydroxybenzoic acid < hydroxycinnamic acids < flavonoid. Hydroxybenzoic derivatives in plants include p-hydroxybenzoic, gallic, syringic, protocatechuic, and vanillic acids which are present as bound components in complex structures like lignins and hydrolysable tannins or attached to cell walls and proteins [23]. Whereas hydroxycinnamic acid derivatives include p-coumaric, ferulic, caffeic, and sinapic acids which occur in bound forms connected to cell wall structures such as cellulose, lignin, sugars, and proteins through ester bonds. Flavonoids are ubiquitous in many plants and most commonly found ones include quercetin and rutin [24]. High content of flavonoids (e.g., anthocyanins, flavones, flavonols and chalcones) may be found in a single structure of plants such as its seeds. Numerous peak overlaps were evident in the fruit extracts suggesting occurrence of similar compounds albeit in various concentrations as indicated by the peak intensities (Figure 7 Panel A).

The KPF extracts demonstrated the strongest peaks which was coherent with the fact that it had the highest TPC and antioxidant capacity values. The higher concentrations of the phenolic compounds in KPF may also explain its strong antibacterial activity determined earlier (Section 3.3). However, it is interesting to note that although KPS show lower concentrations of the phenolic compounds, it was found to be one of the most potent extracts against the cancer cell lines. These discrepancies in the data could mean that the cytotoxic effect of KPS extracts may not be due to the phenolic compounds but rather glycosidic forms of the aglycone phenols that have not been captured in the chromatogram. A previous study has successfully demonstrated that two isolated flavonoid glycosides (2′ -hydroxyl neophellamuretin and 200-O-rhamnosylswertisin) isolated from *Desmodium caudatum* (Thunb.) DC had a certain growth inhibitory effect on HeLa cells in a dose dependent manner [25]. The chromatographic separation, isolation and characterization of glycosylated phenolic compound is often challenging due to their complex structural conformity arising through varying degree of plausible glycosylation patterns and high polarity [26].

Moreover, since GGL and Kakadu plum extracts (KPS and KPF) extracts had shown interesting bioactivity, further peak characterization was warranted. In our previous work we had tentatively identified the peaks of the GGL by comparing their UV spectrums and retention times to that of authentic standards as shown in Figure 7 (Panel C) [3]. A high occurrence of t-ferulic acid in the GGL extracts (Figure 7C) was found which may explain the cytotoxic activity of GGL extracts. Ferulic acid isolated from *Ferula foetida*, which is a perennial herb, has shown anticancer activity against various types of cancers such as colon and lung cancer, and central nervous system tumors previously [27]. Additionally, studies have also demonstrated cytotoxicity of flavonoids against cancer cells and have found them to also have high free radical scavenging activity [28]. Purified flavonoids have shown anticancer activities against a number of human carcinomas which include hepatoma (Hep-G2), cervical carcinoma (Hela), and breast cancer (MCF-7) [28].

**Figure 8:**
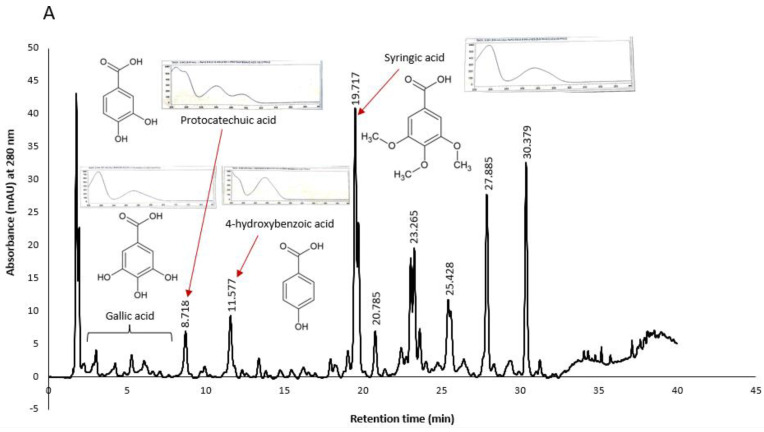

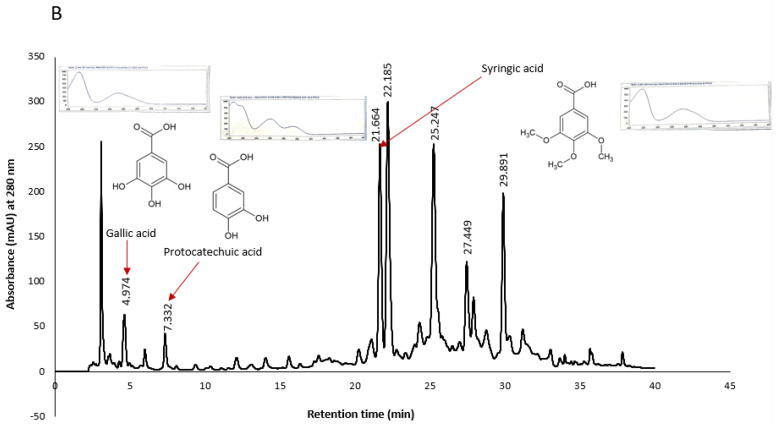
Chromatograms of KPS (**A**) and KPF (**B**) extracts showing tentatively identified peaks based on retention times of standard phenolic compounds.

Similarly, some peaks in KPS and KPF extracts were tentatively identified as shown in Figure 8. It was worth noting that chromatograms of both KPS and KPF extracts measured at a wavelength of 280 nm showed peaks of compounds belonging to the hydroxybenzoic acid class. More specifically both the UV spectral characteristics and retention times of the extract’s peaks closely matched that of gallic acid, protocatechuic acid, 4-hydroxybenzoic acid and syringic acid standards analyzed under similar HPLC conditions.

Konczak et al. (2014) have previously identified hydrolysable tannins and ellagic acids as the major phenolic compounds in Kakadu plum. Kakadu plum belong in the family Combretacea, order Myrtale, which has a significant chemotaxonomic feature which is the presence of ellagic acid and ellagitannins [29]. In this study however, we were unable to identify ellagic acid peaks due to the unavailability of the standard. None the less, the spectral characteristics of the later peaks (RT > 20 min.) match literature UV spectrum of ellagic acid [29,30]. Therefore, it may be presumed that these peaks may be that of ellagic acid. Kakadu plum is unique in that it is the only fruit with the highest ascorbic acid content and that unlike other common fruits, hydroxycinnamic acid and flavonoid compounds are not its major phytochemicals. Further fractionation of Kakadu plum extracts and structural elucidation of compounds for the development of novel antioxidant and/or anticancer drugs is merited.

### 2.5. Cytotoxic Activity of GGL Fractions

**Figure 9:**
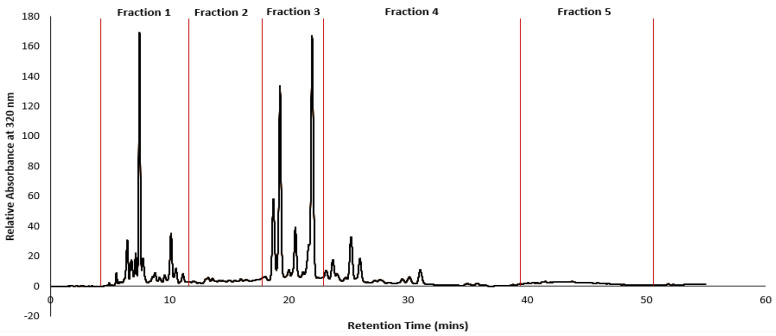
HPLC chromatogram of *P. angustifolium* and retention times at which fractions were collected.

**Figure 10:**
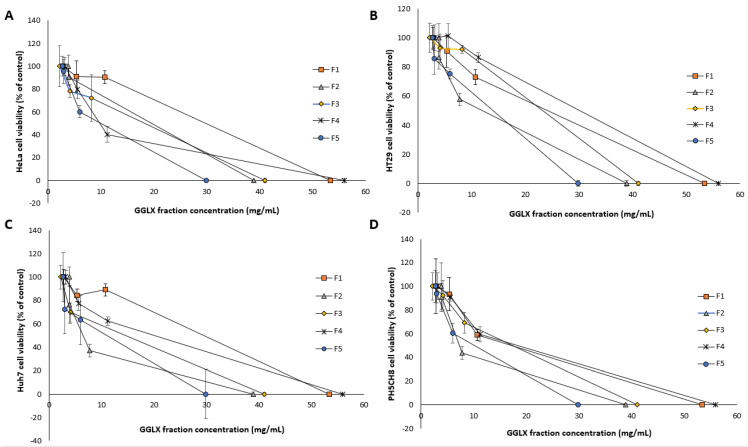
Dose-dependent study of GGL fractions against (**A**) HeLa, (**B**) HT29, (**C**) Huh7 and (**D**) PH5CH8 cell lines.

Given the complete inhibition of cancer cell lines subjected to GGL crude extracts, HPLC time-based fractionation of the extracts were performed as shown in Figure 9 (Phase 2- Bioassay guided fractionation protocol, Figure 1). Dose dependent inhibitory effects of the fractions were determined, and the normalized percentage cell viability data are given in Figure 10. The IC_50_ and SI values of the GGL fractions (F1–F5) against the tested cell lines were calculated and is presented in Table 4.

Highest cytotoxic activities were postulated for Fractions 1 (F1) and Fraction 3 (F3) due to high peak intensities. However, the IC_50_ values of Fraction 2 (F2) was the lowest (0.6–0.92 mg/mL) against the cancer cell lines, deeming it to be most potent. In the F2 time frame of the chromatogram (Figure 9), absence of phenolic peaks suggest that the cytotoxic activity of this fraction may likely be due to other compounds in the matrix rather than polyphenols. Although F2 presented ideal IC_50_ values in comparison to the other extracts, its SI values was quite low (Table 5).

**Figure 11:**
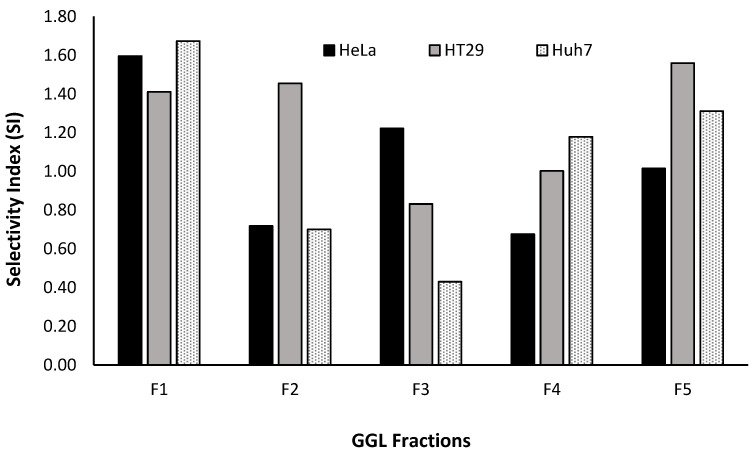
GGL fractions SI values against HeLa, HT29 and Huh7 cell lines.

The SI values of the fractions were graphed as shown in Figure 11 and it was construed that F1, having the highest SI value for HeLa, HT29 and HuH7 (1.60, 1.41 and 1.67, respectively) would be an ideal choice for further fractionation and isolation process (Phase 3- Bioassay guided fractionation protocol, Figure 1). Thus far, the proposed bioassay guided fractionation protocol has successfully demonstrated an effective and robust screening process of the native Australian fruits and GGL samples analyzed. In last decade this technique has been successfully utilized to isolate various effective compounds from plants such as quinine, morphine, paclitaxel, camptothecin, etopoide, mevastin and astemisinin [31].

Previously taraxastane-type triterpene saponins and triterpene glycosides, which have also demonstrated antiproliferative activity have been isolated from GGL [32,33,34,35]. However, due to the occurrence of several varietals of this plant which yield thousands of complex compounds, there still remains research opportunities. Hence, it is anticipated that further fractionation of GGL F1 may possibly lead to the isolation and purification of lead or novel bioactive compound(s).

## 3. Materials and Methods

### 3.1. Reagents

All reagents used were analytical grade or higher purity. Hydrochloric acid and sodium carbonate was purchased from Chem-Supply. All other reagents including the HPLC grade methanol, were purchased from Sigma-Aldrich (Australia). Some reagents used in the cytotoxicity analysis which included the CellTiter 96^®^ AQueous Assay (composed of solutions of tetrazolium compound [3-(4,5-dimethylthiazol-2-yl)−5-(3-carboxymethoxyphenyl)−2-(4-sulfophenyl)−2H-tetrazolium, inner salt; MTS(a)] and an electron coupling reagent (phenazine methosulfate; PMS), commonly known as MTS reagent, and Fetal bovine serum (FBS) were obtained from Promega (United States of America) and Cytiva (United States of America), respectively. The Dulbecco’s Modified Eagle’s Medium—high glucose (DMEM), Dulbecco’s Phosphate Buffered Saline (DPBS) solution were kept in the dark at 4 °C, while the other reagents used in the bioassays were frozen until required for use. All dilutions and assay preparations used Milli-Q water.

### 3.2. Sample Collection and Preparation

**Figure 12:**
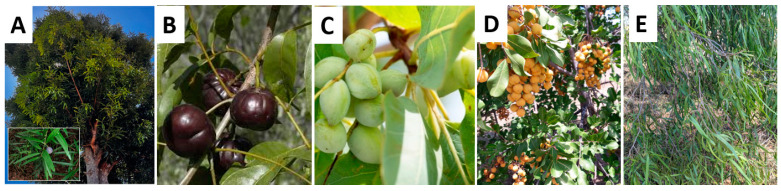
(**A**) *Podocarpus elatus* (Illawarra plum), (**B**) *Pleiogynium timoriense* (Burdekin plum), (**C**) *Terminalia ferdinandiana* (Kakadu plum), (**D**) *Cupaniopsis anacardioides* (Tuckeroo) and (**E**) *Pittosporum angustifolium* (Gumbi gumbi).

The native Australian plant species selected for this study included leaves from *Pittosporum angustifolium* (Gumbi gumbi), fruits from *Terminalia ferdinandiana* (Kakadu plum), *Cupaniopsis anacardioides* (Tuckeroo), *Podocarpus elatus* (Illawarra plum) and *Pleiogynium timoriense* (Burdekin plum). The samples were procured from various locations and is described in Table 5). Images of plants used in this study are given in Figure 12. In case of leaves, 20–50 g of the plant material was sourced. Approximately, 4–6 fruit samples from each species were obtained.

The flesh was separated from the seeds of the fruit samples collected and the gumbi gumbi leaves were separated from the stem. Fresh weights of the samples were taken and subsequently frozen at −80 °C and freeze-dried (−50 °C; 100 mTorr) to gravimetrically determine the moisture content. The dried samples were ground into fine powder using a Breville Coffee & Spice Grinder to obtain a homogenous mixture.

### 3.3. Extraction Protocol and Measurement of TPC and Antioxidant Capacity

Approximately 0.5 g of the finely ground plant samples was extracted with 15 mL of 90% methanol (1:30 *w*/*v*), as described in our previous publications [3,36]. The supernatants from the extractions were collected and stored in dark at 4 °C until required for total phenolic content (TPC) and antioxidant analysis. The TPC was measured using the Folin–Ciocalteu assay and the ferric reducing antioxidant capacity (FRAP) assay was used to measure the antioxidant capacity of the extracts, as described previously [37].

### 3.4. Extraction for Cytotoxicity and Antimicrobial Bioassays

A similar extraction protocol to the previous section was followed, except for upscaling the amounts to 2.5 g of powdered samples in 75 mL of 90% methanol, maintaining the 1:30 *w*/*v*. The supernatant obtained was filtered using 0.45 µm Advantec filter paper and evaporated under reduced pressure at 27 °C to a semi-solid consistency using a rotary evaporator. The semi-solid product was redissolved in approximately 25 mL Milli-Q water and freeze-dried under vacuum (Flexi-Dry Freeze-dryer, −47 °C, 277 mTorr) for 72 h to obtain a fine lyophilized product. The dried product was weighed to determine the percentage yield (Table 5). The lyophilized product was stored at 4 °C in the dark until required for the bioassays. Stock solutions of the plant extracts were prepared by weighing the appropriate amounts and dissolving in known volume of sterilized Milli-Q water. This was followed by sterile filtration (Whatman 0.2 µm PES filter Media) of the stock solution and serial dilutions in DMEM media to obtain required concentrations. Stock solutions of the plant crude crystals were prepared fresh daily as required.

### 3.5. Cytotoxicity Bioassay

The cytotoxicity of the extracts were assessed following our previously published work [3]. Briefly, HeLa (human cervical carcinoma), HT29 (human colorectal carcinoma), HuH7 (human lung carcinoma) and PH5CH8 (human epithelial cell), obtained from the University of Adelaide, were cultured. The cells were maintained as mono layers, until reaching approximately 80% confluency. After this, the cultured flasks were rinsed with DPBS twice, followed by addition of trypsin and incubation for 10 min. to dislodge the cells. Appropriate dilution of the cell suspension was made to obtain a final concentration of 100,000 cells/mL. Aliquots of the 100 µL diluted cell suspension were added to the wells of the 96 well plate (100 µL cells/well) in triplicates. The plate was incubated at 37 °C, 5% CO_2_, for 24 h prior to the addition of 100 µL of plant extracts or cell media (for the negative control) to individual wells. Cisplatin, which is chemotherapeutic drug was used as the positive control. The plates were incubated after the addition of the extracts for a period of 48 h, after which 10 µL of MTS reagent was added to each well. The plated were again incubated for an hour, after which, absorbance reading of the plates were measured 490 and 630 nm using a 96-well BIO-RAD iMark plate reader. This bioassay was repeated three times to test for reproducibility.

Estimation of the half maximal inhibitory concentration (IC_50_) values was performed using an online IC_50_ calculator, AAT Bioquest (https://www.aatbio.com/tools/ic50-calculator; accessed on 10 October 2022) which calculated the IC_50_ values from the treatment concentrations and the corresponding cell viability (%) after background correction (subtracting the smallest response) and normalization (dividing by the largest response). Margin of error (MOE) at 95% confidence interval was calculated based on Equation (1).

(1) $$MOE=t_{crit}\times \frac{SE}{m}\times \sqrt{\frac{1}{n}+\frac{1}{k}+\frac{{\left(y-\bar{y}\right)}^{2}}{SSxx\times m^{2}}}$$

where:

$t_{crit}$ —critical *t*-value which defines the MOE

SE —standard error of regression

*m*—gradient of regression at point of evaluation (*y*-values, i.e., percentage cell inhibition)

*n*—number of observations (*x*-values, i.e., the log concentrations)

*k*—number of replicates

*y*—evaluation points (*y*-value)

$\bar{y}$ —mean of y-values

SSxx —sum of squares of the *x*-values

The efficacy of the extracts against normal cells (PH5CH8) was determined by calculating the selectivity index (*SI*), which is the ratio of the toxic concentration of the extract against its effective bioactive concentration as shown in Equation (2).

(2) $$SI=\frac{IC_{50}\;against\;PH5CH8\;cells\;\left(toxic\;concentration\right)}{IC_{50}\;against\;cancer\;cell\;lines\;}$$

#### Flow Cytometry Analysis

Cells with compromised cell membranes (dead or dying cells) will uptake the 7-AAD (7-Aminoactinomycin D) stain, fluorescing when analysed via flow cytometry. Since the purpose of this analysis was to check cell viability of the cells subjected to the plant extracts, similar steps to the MTS assay were followed with slight modifications. On day 4, the cells were dislodged from the 96 well plates using trypsin (Plate A) and transferred to the respective wells in another round bottom 96 well plate (Plate B) and centrifuged at 1300 rcf for 15 min. The supernatant was removed, and the cells were resuspended in 200 µL of 7-AAD solution (2 µg/mL in PBS) and transferred into 2 mL Eppendorf tubes. The tubes were then incubated in the dark for 10 min. and then analyzed on a BD FACSVerse flow cytometer (BD Biosciences Australia). The population has been gated to exclude debris and dead cells as described in previous literature [12].

### 3.6. Antimicrobial Activity

Four bacterial strains commonly associated with food poisoning diseases were selected to test the antibacterial potency of the test samples (plant extract). One strain of Gram positive (*Staphylococcus aureus*) and three strains of Gram negative (*Escherichia coli*, *Salmonella typhi* and *Pseudomonas aeruginosa*) bacteria. The bacterial strains were provided from the culture collection of School of Health, Medical and Applied Sciences, Central Queensland University, Australia. Pure cultures of the bacteria were plated on agar plates using the streaking method and incubated at 37 °C for 48 h.

An isolated colony from each bacterial strain was swabbed from the streaked plates and sub-cultured in 20 mL of Muller Hinton Broth (MHB) to attain viable cell count of approximately 10^7^ CFU/mL (estimate based on the plate count in the previous section). The inoculum was incubated at 37 °C for 24 h. The prepared inoculum (approx. 10^7^ CFU/mL) was again diluted in MHB to obtain an approximate concentration of 10^5^ CFU/mL. The diluted inoculum (0.1 mL) was added to 25 mL of Muller Hinton Agar MHA (melted at 53 °C until the solution became clear). The MHA seeded with the inoculum was then poured into the plate and allowed to set.

The disc diffusion method [38] with slight modification was used to evaluate the antimicrobial activity of each plant extract against the four bacterial strains. The plant extract crystals (20 mg) were redissolved in 1 mL of sterile Milli-Q water, sterilized through Whatman 0.2 µm PES filter Media. Sterile filter paper discs (8 mm diameter) were placed on top of the MHA plates and then loaded with 10 µL of the plant extracts (0.2 µg of the plant crystals on the disc). Filter paper discs loaded with 5 µg of Gentamycin was used as the positive control. The plates were refrigerated (5 °C) for 2 h to allow plant extract diffusion then incubated at 37 °C for 24 h. The zones of inhibition were measured by Vernier caliper and recorded.

### 3.7. High Performance Liquid Chromatography (HPLC) Phenolic Profiling and Fractionation

The phenolic profile of the freeze-dried extract used in the bioassays was performed using a HPLC protocol developed in our laboratory previously [3] with slight modifications. Briefly, an Agilent 1100 HPLC system with C-18 reversed phase column and multiwavelength detector module was used. The mobile phase comprised 90% methanol (A) and 0.01 M phosphoric acid (B). Gradient elution as given in Table 6 was followed, with a flow rate of 1 mL/min and an injection volume of 5 µL. The total run time was 50 min., with a post-run flush time of 10 min. With reference to retention time, the polyphenols present in the extracts were classified as hydroxybenzoic acid < hydroxycinnamic acids < flavonoid. Using UV spectral data of some commonly occurring plant phenols, the peaks on the HPLC profiles of the plant extracts were tentatively identified. The HPLC was connected to an Agilent 1260 Infinity II Analytical-Scale fraction collector (G1354F). Since the *P. angustifolium* crude extracts had initially shown the most promising bioactivity, it was selected for fractionation. Five fractions were collected based on the retention times of predominant peaks, between 5.50 to 10.00, 10.00 to 19.00,19.00 to 24.50, 24.50 to 35 and 35.00 to 50.00 min.

### 3.8. Cytotoxicity of Gumbi Gumbi Leaves (GGL) Fractions

All fractions were rotary evaporated down to semi-solid fraction and redissolved in 25 mL Milli-Q water, frozen at −80 °C and freeze-dried (−47 °C, 50 mTorr). After weighing the fraction residues (50–76 mg) it was dissolved in 20 mL sterile Milli-Q water and sterile filtered through Whatman 0.2 µm PES filter Media. Cytotoxicity assay of the fractions were performed as mentioned in the earlier sections. The IC_50_ and SI values were applied as the exclusion criteria for fractions.

### 3.9. Statistical Analysis

All data were exhibited as means ± SEM for triplicate sample analysis. In the case of the bioassays, this comprised at least three independent experiments. Utilizing RStudio (running version R-4.2.1 (2022–06–23), Robert Gentleman and Ross Ihaka, University of Auckland), statistical analysis was performed with a one-way analysis of variance to determine statistical significance between control and treated groups with a value of *p* < 0.05 considered to be statistically significant.

## 4. Conclusions

The study of native Australian plants is important to not only understand their nutritional value but also their therapeutic potential. This study was unique as it was the first to compare the phytochemical content and bioactivity of water-soluble products from five different native plants. This study also presented a novel bioassay guided fraction protocol design which was efficient and effective in screening out GGL extracts as a promising therapeutic plant and identifying the fraction of GGL extracts that may have promising novel and safe anticancer compound(s). The highest values were found in the KPF which were 20,847 ± 2322 mg GAE/100 g TPC and 100,494 ± 9487 mg TXE/100 g antioxidant capacity. Extracts of GGL was deemed to be most potent with complete cell inhibition in HeLa and HT29, and about 95% inhibition in HuH7 cells. Comparative activity was also seen for KPS extract, where more than 80% cell inhibition occurred in all tested cell lines. Dose-dependent studies showed higher SI values (0.72–1.02) in KPS extracts than GGL (0.5–0.73). KPF extracts were effective in suppressing microbial growth of all tested bacterial strains except for *P. aeruginosa*, while TKS and TKF were only slightly effective against *S. aureus.* Due to the potential of the GGL crude extract to completely inhibit the cells compared to KPS, it was further fractionated and tested against the cell lines. Other plant extracts showed no antibacterial activity against the pathogenic bacterial strains. HPLC phenolic profiling of the crude extracts were performed, and numerous peak overlaps were evident in the fruit extracts suggesting occurrence of similar compounds albeit in various concentrations as indicated by the peak intensities. The KPF extracts demonstrated the strongest peaks which was coherent with the fact that it had the highest TPC and antioxidant capacity values. A high occurrence of t-ferulic acid in the GGL extracts was found which may explain the cytotoxic activity of GGL extracts. Peaks in KPS and KPF extracts were tentatively identified as gallic acid, protocatechuic acid, 4-hydroxybenzoic acid and syringic acid and possibly ellagic acid. HPLC time-based fractionation of the GGL extract (F1–F5) was performed and dose dependent cytotoxic effects were determined. It was construed that F1, having the highest SI value for HeLa, HT29 and HuH7 (1.60, 1.41 and 1.67, respectively) would be promising for further fractionation and isolation process, which is phase 3 of the bioassay guided fractionation protocol. Some interesting data on KPF and KPS in terms of their antibacterial and anticancer activity, respectively, were also found and further studies on their fractions will also be conducted. Finally, more studies of Australian native plants are warranted as it will promote the use of these plants as food, in pharmacology and potentially enable sustainable Indigenous agricultural businesses to be created.

## Figures and Tables

**Table 1:** Average TPC and antioxidant capacity of the plant extracts.

Plant Samples	Avg TPC (mg GAE/100g)	Avg FRAP (mg TXE/100 g)
TKF	9085 ± 393 ^b^	12,351 ± 1905 ^d^
TKS	2686 ± 15 ^c^	1181 ± 56 ^bcd^
BPF	12,442 ± 1355 ^b^	16,670 ± 2275 ^bc^
BPS	2647 ± 38 ^c^	4484 ± 308 ^cd^
IPF	4193 ± 20 ^c^	5705 ± 247 ^cd^
IPS	2764 ± 690 ^c^	1966 ± 311 ^d^
KPF	20,847 ± 2322 ^a^	100,494 ± 9487 ^a^
KPS	2927 ± 208 ^c^	23,511 ± 1192 ^b^
GGL	4169 ± 57 ^c^	6742 ± 923 ^cd^

One-way ANOVA test indicated statistical significance (*p*-value < 0.05) between plant extracts, denoted by different letters. All values are given as means ± 2 SD (*n* = 3).

**Table 2:** GGL and KPS crude extract IC_50_ and SI values against tested cell lines.

	GGL			
	HeLa	HT29	Huh7	PH5CH8 (Normal Cell)
IC_50_ * (µg/mL)	472.15	687.64	ND	344.83
Log IC_50_	2.67	2.84	ND	2.54
MOE **	−0.24	−0.29	ND	−0.28
95% CI *** of Log IC_50_	2.44–2.91	2.55–3.13	ND	2.26–2.82
SI	0.73	0.5	ND	
	**KPS**			
	**HeLa**	**HT29**	**Huh7**	**PH5CH8** (**Normal Cell**)
IC_50_ * (µg/mL)	420.07	296.81	363.38	302.19
Log IC_50_	2.62	2.47	2.56	2.48
MOE **	−0.23	−0.20	−0.20	−0.20
95% CI *** of Log IC_50_	2.39–2.85	2.27–2.68	2.36–2.76	2.57–2.97
SI	0.72	1.02	0.83	

* AAT Bioquest Calculated value; ** MOE—Margin of Error; *** CI—confidence interval.

**Table 3:** Average zone of inhibition (*n* = 3) of the crude plant extracts at a concentration of 20 mg/mL.

Plant Extracts	Bacterial Strain Zone of Inhibition (mm)			
	Gram Positive	Gram Negative		
	*S. aureus*	*E. coli*	*P. aeruginosa*	*Sal. typhimurium*
TKF	8.5 ± 0.71	0.00	0.00	0.00
TKS	9.25 ± 0.35	0.00	0.00	0.00
BPF	0.00	0.00	0.00	0.00
BPS	0.00	0.00	0.00	0.00
IPF	0.00	0.00	0.00	0.00
IPS	0.00	0.00	0.00	0.00
KPF	13.40 ± 1.13	15.70 ± 0.42	0.00	11.85 ± 2.05
KPS	0.00	0.00	0.00	0.00
GGL	0.00	0.00	0.00	0.00
Positive control (gentamicin)	27.75 ± 0.35	22.75 ± 0.35	26.75 ± 0.35	22.25 ± 0.35

All values are given as means ± 2 SD (*n* = 3).

**Table 4:** GGL fractions inhibitory concentrations and SI values.

GGLX Fractions	F1	F2	F3	F4	F5
HeLa Inhibitory Conc. (mg/mL)	19.1	4.07	14.12	8.77	6.4026
Log IC_50_	1.28	0.61	1.15	0.94	0.81
MOE	−0.34	−0.19	−1.47	−0.16	−0.91
95% CI of Log IC_50_	0.94–1.62	0.42–0.80	−0.32–2.62	0.78–1.60	0.73–0.88
SI	1.60	0.72	1.22	0.68	1.01
HT29 Inhibitory Conc. (mg/mL)	16.89	8.25	9.61	13	9.84
Log IC_50_	1.23	0.92	0.98	1.11	0.99
MOE	−0.23	−0.18	−0.35	−0.9	−0.67
95% CI of Log IC_50_	1.00–1.46	0.73–1.10	0.63–1.34	0.21–2.02	0.32–1.66
SI	1.41	1.46	0.83	1.00	1.56
Huh7 Inhibitory Conc. (mg/mL)	20.03	3.97	4.97	15.28	8.27
Log IC_50_	1.3	0.6	0.7	1.18	0.92
MOE	−0.34	−0.31	−0.26	−0.16	−0.91
95% CI of Log IC_50_	0.96–1.64	0.29–0.91	0.44–0.95	0.98–1.38	0.00–1.83
SI	1.67	0.70	0.43	1.18	1.31
PH5CH8 Inhibitory Conc. (mg/mL)	11.97	5.67	11.56	12.97	6.31
Log IC_50_	1.08	0.75	1.06	1.11	0.8
MOE	−0.22	−0.2	−0.28	−0.15	−0.07
95% CI of Log IC_50_	0.86–1.30	0.55–0.95	0.78–1.35	0.96–1.27	0.73–0.87

**Table 5:** Percentage yield of crude crystal product.

Sample Name	Location	Traditional Use	% Recovered
Kakadu plum flesh (KPF)	Charles Darwin University, Northern territory.	Remedy for a cold or flu.	37.9
Kakadu plum seed (KPS)			18.3
Burdekin plum flesh (BPF)	North Rockhampton, Central Queensland.	Used in jellies, jams and as preservatives. Flavor meat or fermented into wine.	42.9
Burdekin plum seed (BPS)			10.4
Tuckeroo flesh (TKF)	Parkhurst, Rockhampton, Central Queensland.	Cure stomach-aches, diabetes and insomnia [21]	7.3
Tuckeroo seed (TKS)			21.4
Illawarra plum flesh (IPF)	Rockhampton, Central Queensland.	Regarded as one of the bush foods	57.0
Illawarra plum seed (IPS)			21.2

**Table 6:** HPLC Gradient elution profile.

Time (min.)	% Solvent A	% Solvent B
0	5	95
2	20	80
12	30	70
18	40	60
35	50	50
45	80	20
50	100	0

## Data Availability

Not applicable.
