# Correction: Mani et al. Bioassay Guided Fractionation Protocol for Determining Novel Active Compounds in Selected Australian Flora. *Plants* 2022, *11*, 2886

**DOI:** 10.3390/plants13213031

**Published:** 2024-10-30

**Authors:** Janice Mani, Joel Johnson, Holly Hosking, Beatriz E. Hoyos, Kerry B. Walsh, Paul Neilsen, Mani Naiker

**Affiliations:** 1 School of Health, Medical and Applied Sciences, Central Queensland University, Bruce Hwy, North Rockhampton, QLD 4701, Australia; 2 Institute of Future Farming Systems, Central Queensland University, Bruce Hwy, North Rockhampton, QLD 4701, Australia

In the original publication [1], there was a mistake in Figure 2 as published. Part of the information in the figure was cut off. The corrected Figure 2 appears below.

There was a mistake in Figure 12 as published. The image of one of the plant samples (A—*Podocarpus elatus*) was incorrect. The corrected Figure 12 appears below.

The values of TPC and FRAP in the text pertaining to KPF were incorrect as per Table 1. A correction has been made to the fifth sentence of abstract and fourth sentence of conclusion section. The correction is as follows: “The highest values were found in the KPF which were 20,847 ± 2322 mg GAE/100 g TPC and 100,494 ± 9487 mg TXE/100 g antioxidant capacity”. 

The authors state that the scientific conclusions are unaffected. This correction was approved by the Academic Editor. The original publication has also been updated.

## Figures and Tables

**Figure 2:**
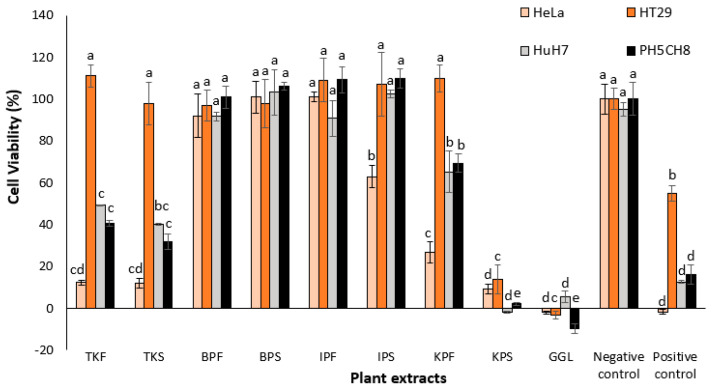
Percentage cell viability of cell lines treated with plant extracts at 500 µg/mL concentration. One-way ANOVA test indicated a significant difference (*p*-value < 0.05) in cytotoxicity between the different plant extracts for the same cell line, denoted by different letters on the respective bars. Negative control: cells without treatment; positive control: cells treated with 50 µg/mL cisplatin (chemotherapy drug).

**Figure 12:**
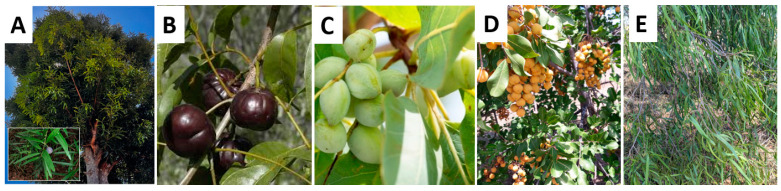
(**A**) *Podocarpus elatus* (Illawarra plum), (**B**) *Pleiogynium timoriense* (Burdekin plum), (**C**) *Terminalia ferdinandiana* (Kakadu plum), (**D**) *Cupaniopsis anacardioides* (Tuckeroo) and (**E**) *Pittosporum angustifolium* (Gumbi gumbi).
